# Estimating colony sizes of emerging bats using acoustic recordings

**DOI:** 10.1098/rsos.160022

**Published:** 2016-03-09

**Authors:** Laura N. Kloepper, Meike Linnenschmidt, Zelda Blowers, Brian Branstetter, Joel Ralston, James A. Simmons

**Affiliations:** 1 Department of Biology, Saint Mary’s College, Notre Dame, IN 46556, USA; 2 Department of Neuroscience, Brown University, 185 Meeting Street, Providence, RI 02912, USA; 3 National Marine Mammal Foundation, 2240 Shelter Drive #204, San Diego, CA 92106, USA

**Keywords:** bats, acoustic monitoring, population census

## Abstract

The decline of bats demands more widespread monitoring of populations for conservation and management. Current censusing methods are either prone to bias or require costly equipment. Here, we report a new method using passive acoustics to determine bat count census from overall acoustic amplitude of the emerging bat stream. We recorded the video and audio of an emerging colony of Mexican free-tailed bats from two cave locations across multiple nights. Instantaneous bat counts were calculated from the video frames, and the bat stream’s acoustic amplitude corresponding to each video frame was determined using three different methods for calculating acoustic intensity. We found a significant link between all three acoustic parameters and bat count, with the highest *R*^2^ of 0.742 linking RMS pressure and bat count. Additionally, the relationship between acoustics and population size at one cave location could accurately predict the population size at another cave location. The data were gathered with low-cost, easy-to-operate equipment, and the data analysis can be easily accomplished using automated scripts or with open-source acoustic software. These results are a potential first step towards creating an acoustic model to estimate bat population at large cave colonies worldwide.

## 1. Background

Bats are on the decline. The widespread mortality of bats across North America due to white-nose syndrome, wind turbine collisions, and habitat loss due to urbanization and agricultural expansion is predicted to result in agricultural losses of billions of dollars per year [1]. There is an urgent need for bat censusing to determine key populations for management and conservation.

Historically, common means of bat censusing included visual estimation [2,3], photographic estimation [4,5], mark recapture [6] and thermal imagery [7–10]. All of these prior methods pose some disadvantage: visual, photographic and mark–recapture techniques are prone to bias [11], and thermal imagery requires expensive cameras and sophisticated tracking programmes [9]. For many researchers and conservation managers, the cost of thermal imaging systems is the biggest limitation for obtaining ongoing, accurate population counts.

Passive acoustic monitoring provides a low-cost and rapid means for monitoring vocalizing animals. Because the equipment is easily deployed and relatively inexpensive, acoustic monitoring has been used to identify species composition and/or relative species abundances in birds [12], frogs [13], insects [14], marine mammals [15] and bats [16]. Despite its advantages, one limitation of passive acoustics is that it can only give an index of abundance. Although understanding species composition and/or relative abundance is important for many management and conservation considerations, the need for accurate population counts persists. Here, we introduce a new method using acoustics to estimate the population size of bat colonies using low-cost, user-friendly equipment.

## 2. Methods

**Figure 1.**
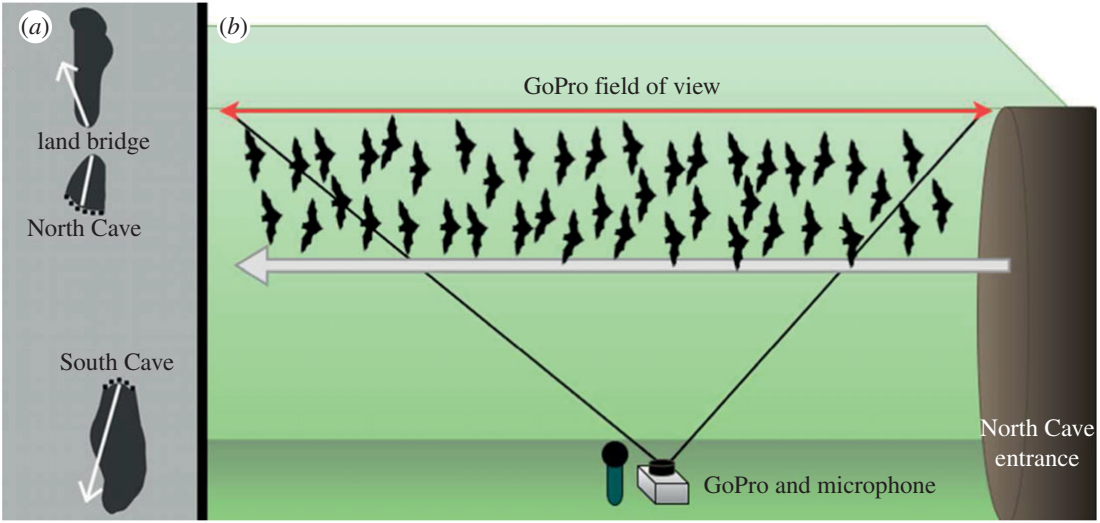
(*a*) Location and orientation of North and South Cave (overhead view). White arrows indicate the flight path of the emerging bat stream. (*b*) Position of audio and video recording equipment relative to the emerging bat stream.

Data were collected from 12 June to 22 June 2015, at a lava tube cave structure located on private land in Sierra County, New Mexico. The field site consisted of two caves: the ‘North Cave’ and the ‘South Cave’ (figure 1*a*), and each housed approximately 700 000–900 000 Mexican free-tailed bats (*Tadarida brasiliensis mexicana*). This rough estimate was achieved by using video analysis to obtain exact bat counts while light levels were sufficient, determining a baseline number, then extrapolating a rough percentage of the baseline number using thermal imagery for the entire emergence.

Each night beginning approximately 20 min before bat emergence, synchronized acoustic (250 kHz, Ultramic-250, Dodotronics, Castel Gandolfo, Italy) and video (240 frames per second, GoPro Hero3+, Black edition, San Mateo, CA, USA) recordings began at both cave locations. Both the camera and microphone were placed below the emerging stream, located adjacent to each other, and pointed at the sky (figure 1*b*).

Each night of the recordings, emergence began approximately between 19.15 and 19.45, peaked in density 5–6 min after onset, and continued for approximately 90 min, extending past dark. For each night and cave location, beginning with the first bat to emerge and continuing until bats could not be distinguished from the dark sky, a video frame and its corresponding 1 s audio file were extracted every 10 s. Bat counts were extracted from video frames using ‘Find the Maxima’ function and batch process in ImageJ. Three acoustic parameters, root mean square (RMS) pressure (in dB), peak-to-peak pressure (in dB) and total energy were calculated for each 1 s sound file according to the following:

RMS pressure = 20 × log_10_(s.d.(waveform));
peak-to-peak pressure = 20 × log_10_(max(waveform) − min(waveform));
total energy = (sum(waveform^2^));
where s.d. = standard deviation, waveform = the voltage measurements in the 1 s sound file, max = maximum, min = minimum.

Owing to atypical flight behaviour with the beginning of the emerging stream, we only analysed the parts of the emergence when bat count was greater than 10 bats.

We fit simple linear regression models with bat counts as the response variable and each of the three acoustic parameters as predictor variables. For RMS pressure and peak-to-peak pressure models, bat count data were log transformed prior to model fit. The acoustic parameter (AP) with the best model fit (largest *R*^2^-value) was used for remaining analyses. We used an ANCOVA to test for homogeneity of regression across caves, using the significance of an AP by cave interaction to determine whether the relationship between AP and bat count varied across caves. We then used a reciprocal cross-validation technique where the fit of cave-specific linear regression models was tested using data from the other cave. Lastly, we estimated total sampled bat counts (TSBC, from the visible portion of the emergence) for each day and cave by separately summing bat counts from video frames and linear model estimates, and compared the means using paired *t*-tests with *p*-values adjusted with a Bonferroni correction for multiple comparisons. Detailed methods and example emergence video are available from the electronic supplementary material.

## 3. Results

Linear regression models with each of the three acoustic parameters predicted bat count data well (RMS pressure: *R*^2^=0.742; peak-to-peak pressure: *R*^2^=0.504; total energy: *R*^2^=0.562). Owing to best fit, the RMS pressure model was used for further analysis. Although there was a positive linear relationship between population and all three acoustic parameters (electronic supplementary material, figure S1), a multiple regression was not used since the acoustic parameters were highly correlated (all pairwise *r*>0.65, *p*<0.001).

**Figure 2.**
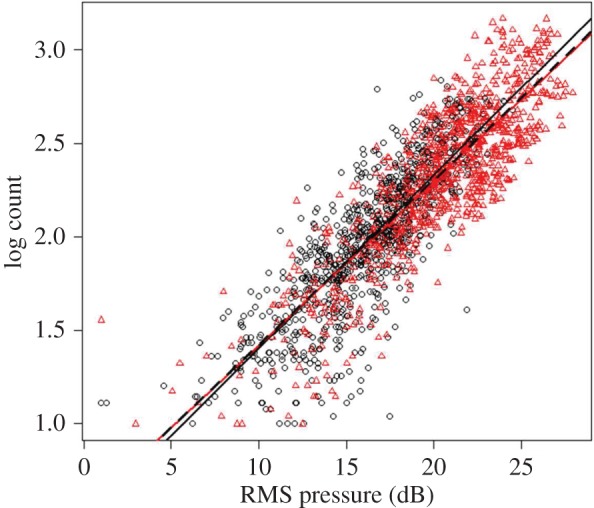
Relationship between RMS pressure (dB) and bat count (logarithm). Black circles: North Cave, grey (red online) triangles: South Cave. The solid black line is the regression fit for the North Cave, and the solid grey line (red online) is the regression fit for the South Cave. The dashed black line is the regression line fit for the entire dataset (North and South combined).

An ANCOVA indicated a non-significant interaction between cave and RMS pressure (*p*=0.074), suggesting the relationship between RMS pressure and bat count does not vary across caves (figure 2). Linear models fit the data from each cave well (*R*^2^_North_=0.659; *R*^2^_South_=0.712), and reciprocal cross-validation demonstrated that the models predicted the bat count at the other caves (North model predicting South data: *R*^2^=0.548; South model predicting North data: *R*^2^=0.495). Averaging counts across all recording days demonstrated good agreement between the video counts and acoustic models. The mean TSBC for both caves did not significantly differ between the video counts or either acoustic model (table 1).

**Table 1.** TSBC for each day and each cave. N = North Cave estimates and S = South Cave estimates. Subscripts indicate the method of estimation: v subscripts represent estimates from video frames; N and S subscripts represent estimates from North and South fit models, respectively. s.e. is standard error. *p*-values represent *t*-test comparisons between each acoustic model and video count.

day	*N*_v_	*N*_N_	*N*_S_	*S*_v_	*S*_N_	*S*_S_
1	16 427	15 560	14 656	n.a.	n.a.	n.a.
2	8165	8574	8404	24 064	17 990	16 630
3	15 886	15 087	14 302	67 163	86 933	76 311
4	13 693	13 966	13 240	29 322	29 813	27 248
5	11 515	7642	7316	32 498	26 478	24 052
6	8408	10 145	9514	24 150	49 200	43 216
7	8455	10 210	9285	45 827	44 954	40 088
8	14 430	8982	8416	46 548	27 035	24 604
9	20 823	13 987	12 886	36 324	26 101	22 985
mean ± s.e.	13 089±1448	11 573±1020	10 891±949	38 237±5139	38 563±7823	34 392±6770
*p*-value	—	0.737	0.289	—	1.00	1.00

## 4. Discussion

This study demonstrates, to the best of our knowledge, the first successful use of passive acoustics to estimate bat counts. We determined a significant relationship between acoustic pressure and individual count at one cave location, and then accurately used the acoustic model to predict the count at another cave location. The data were gathered with low-cost (approx. $800 USD total), easy-to-operate equipment, and the data analysis can be easily accomplished using automated scripts (such as MATLAB or R) or with open-source acoustic software. These results are a potential first step towards creating a comprehensive acoustic model to estimate bat population at large cave colonies worldwide.

There was a significant linear relationship between RMS pressure and the logarithm of bat count for both cave locations, and the model trained at one cave could successfully predict the number of counted bats at another cave using only acoustic information. ANCOVA models for both caves had a significant interaction between AP and day, indicating the slope of a regression line for the relationship between AP and bat count varied across days for both caves (electronic supplementary material, figures S2 and S3). Although Mexican free-tailed bats can demonstrate daily fluctuations in colony size and time of emergence [5], we believe this interaction between AP and day is due to variation in the sample size and distribution of data across days. Note that while the variation around the mean TSBC may appear large (table 1), they result from a different proportion of the population being counted each night. For example, on some days bats emerged in fewer events with higher densities, while on other days emergences were more variable in the density (table 1). These differences suggest data should be pooled across days to ensure adequate sampling across a range of AP values.

It is important to acknowledge four limitations of our study. First, this method can only successfully be applied to emergence rates of 10 bats per second or greater. Correlation values between AP and bat count for values less than 10 bats are very low, which is probably due to a circling behaviour (flying back and forth) of bats at lower emergence densities at our field site. Therefore, this method can only be applied to locations where bats emerge from their roost in a linear fashion. Further testing of this method at additional colony sites should determine whether this limitation persists at other sites. Second, the cave entrances in this study were in open areas. Many bat roosts are surrounded by vegetation, including tree branches, which may change the stream intensity and affect the relationship between stream intensity and emergence rates. Third, this method was used on a cave with a single species of bat. Because different species can make different call parameters, this method needs to be tested with a mixed-species roost. Fourth, our study was limited to the visible part of emergence, which corresponds to approximately one-third of the duration of the total emergence. Thermal monitoring, however, demonstrated that the visible portion of emergence represented the largest range of bat counts, so we expect the relationship between acoustics and bat count to persist for the entire emergence period. Additionally, since our results demonstrate that the acoustic models can successfully be extrapolated beyond the data they were trained on (table 1), this method can be applied to acoustic data from bats emerging at dark. In other words, if the regression equation between acoustics and population can be established using video from the visible part of the emergence, the entire colony population can be estimated by applying the regression equation to the acoustic recording of the entire emergence. For colonies that emerge entirely in the darkness, low-cost (<$5000 USD) thermal imagery can replace the GoPro video camera to obtain population counts to link to acoustics.

In order to use this method to determine the entire colony population, a few additional steps are needed. First, we sampled one video frame and one 1 s audio clip every 10 s. This assumes bats cross the entire camera’s field-of-view in 1 s. For accurate population census, this assumption should be verified and/or a correction factor applied. For example, we determined bats cross our field-of-view in an average of 0.7 s. Therefore, to accurately estimate bat population we would multiply our bat count for each video frame by 1.43 (1/0.7). If the entire emergence were recorded, we could then sum up each observation and multiply it by the sampling interval (in our case, by 10). Additionally, at both of our recording sites, the bats were flying the same distance above the microphone. Since acoustic pressure decays with distance, further studies would need to account for differential heights of emerging bats in the acoustic parameters.

Our findings demonstrate the first important step towards creating a passive acoustics model for bat censusing that is affordable, reliable, and easy to operate and analyse. The next steps include similar recordings at caves of different species compositions, bat densities and topographies. With further replication of our recordings at other sites, we can determine additional parameters that might influence the relationship between acoustics and bat count, as well as quantify the degree of uncertainty surrounding each population estimate. The ultimate goal is to refine our methods to develop a model that could be applied to any bat colony location. This method could be used to monitor bat colonies and determine critical sites for conservation and management.

## Supplementary Material

Supplemental_Information

## Supplementary Material

Figure S1

## Supplementary Material

Figure S2

## Supplementary Material

Figure S3

## Data accessibility

Data is available at http://dx.doi.org/10.5061/dryad.td03h.
