# Supplementary material for: Estimating colony sizes of emerging bats using acoustic recordings
Source: R Soc Open Sci. 2016 Mar 9;3(3):160022. doi: 10.1098/rsos.160022 (PMC4821278; doi:10.1098/rsos.160022)
Supplement: Supplemental_Information [file rsos160022supp1.docx]

Supplemental Information

Expanded methods:

Recording site

Data were collected from June 12-June 22, 2015, at a lava tube cave structure located on private land in southern New Mexico in the Chihuahuan Desert ecoregion. The lava tube had collapsed in sections creating a canyon and two cave openings (Figure 1A, main article). The first, termed the "North Cave," opened to the north into a canyon. This canyon continued for approximately 60 m, was interrupted by a 30 m land bridge, and then opened again for another 120 m. The second cave, termed the "South Cave," was located 200 m south of the North Cave and opened south directly into a canyon that was 120 m long. Each night of the recordings, emergence began at approximately 7:30 pm, peaked in density 5-6 minutes after onset, and continued for approximately 90 minutes, extending beyond dark.

Recording equipment

Each night beginning approximately 20 minutes before emergence, acoustic and video recordings began at both cave locations and the audio and video equipment were synchronized. Continuous video was recorded at 240 frames per second using an action camera (GoPro Hero3+, Black edition, San Mateo, CA). This camera was placed below the stream and pointed towards the sky, which provided good contrast in the twilight (Video V1). Echolocation sounds were recorded using an ultrasonic microphone (Ultramic-250, Dodotronics, Castel Gandolfo, Italy) sampled at 250 kHz with the USB Audio Recorder PRO software (eXtream Software Development, The Netherlands) and stored on both a Samsung Galaxy S3 phone and a Samsung Galaxy TabPro tablet (Samsung, Ridgefield Park, New Jersey, USA) as wave files. The microphone was positioned adjacent to the action camera and pointed at the sky. Both the microphone and action camera were placed in the same position and orientation each night.

Video and sound analysis

For each night and cave location, beginning with the first bat to emerge and continuing until dark, a video frame was extracted every 10 seconds. Using ImageJ, the number of bats present in that video frame was counted with the "Find the Maxima" function and batch process. Then, a 1-second long audio recording corresponding to the video frame was extracted. This audio recording was filtered using an 8^th^ order Butterworth Highpass filter at 15 kHz. The root-mean-square (RMS) pressure (in dB), Peak-to-peak pressure (in dB), and total energy within the recording were calculated using a custom MATLAB algorithm. For each 1-second sound file (waveform), the three acoustic parameters were calculated as follows:

RMS pressure = 20*log10(std(waveform));

Peak-to-peak pressure = 20*log10(max(waveform)-min(waveform));

Total energy = (sum(waveform.^2));

Supplemental Figures


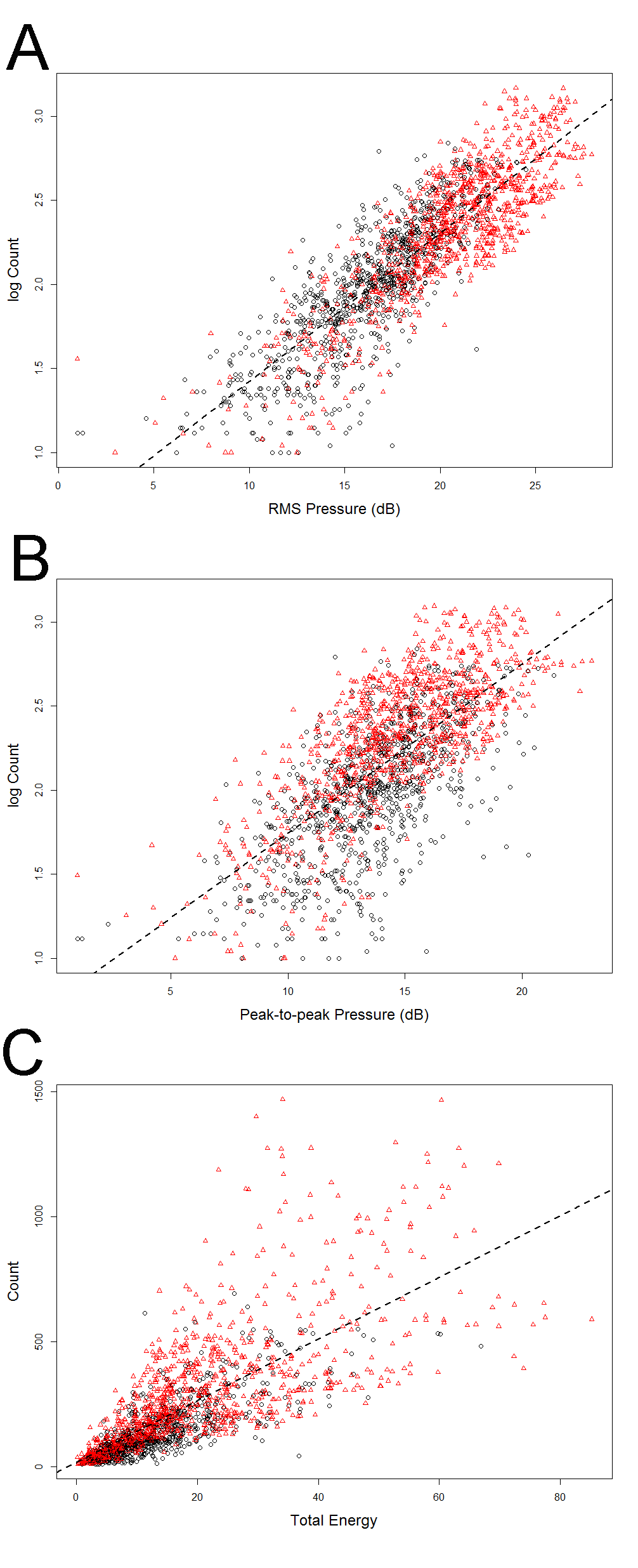


Figure S1: Relationship between acoustic parameter and emergence count for different measured acoustic parameters. Black circles indicate data from the North Cave, and red triangles indicate data from the South Cave. The dashed black line represents the regression model for both caves combined. (A) Bat count (log) as a function of RMS pressure (AP1). Log Count = 0.088*AP1 + 0.537 ;  R-squared:  0.7424

(B) Bat count (log) as a function of peak-to-peak pressure (AP2). LogCount = 0.101*AP2 + 0.739; R-squared: 0.504 (C) Bat count as a function of Total Energy (AP3). Count = 12.328AP3 + 16.0938; R-squared: 0.5615


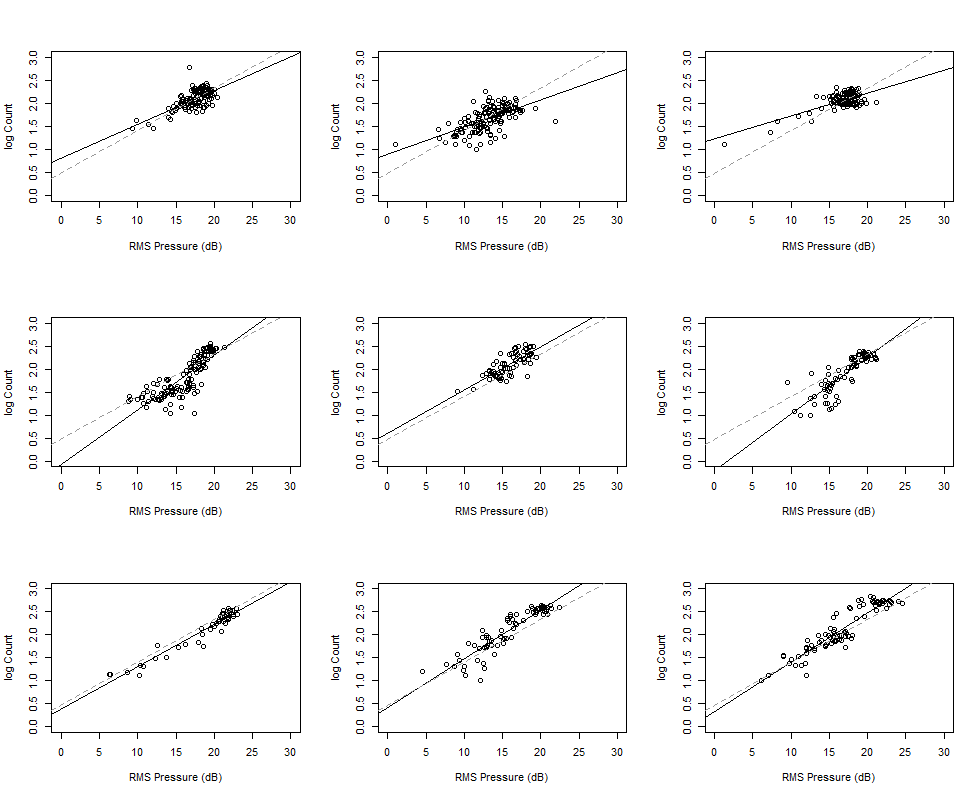


Figure S2. Comparison of RMS pressure to log count for each recorded night at the North Cave. The solid black line represents the regression model for each day, and the dashed line represents the regression model for all days combined (i.e. solid black line in Figure 1 in the main article). The distribution of emergence density varied across nights, potentially leading to the AP*day interaction in ANCOVA models.


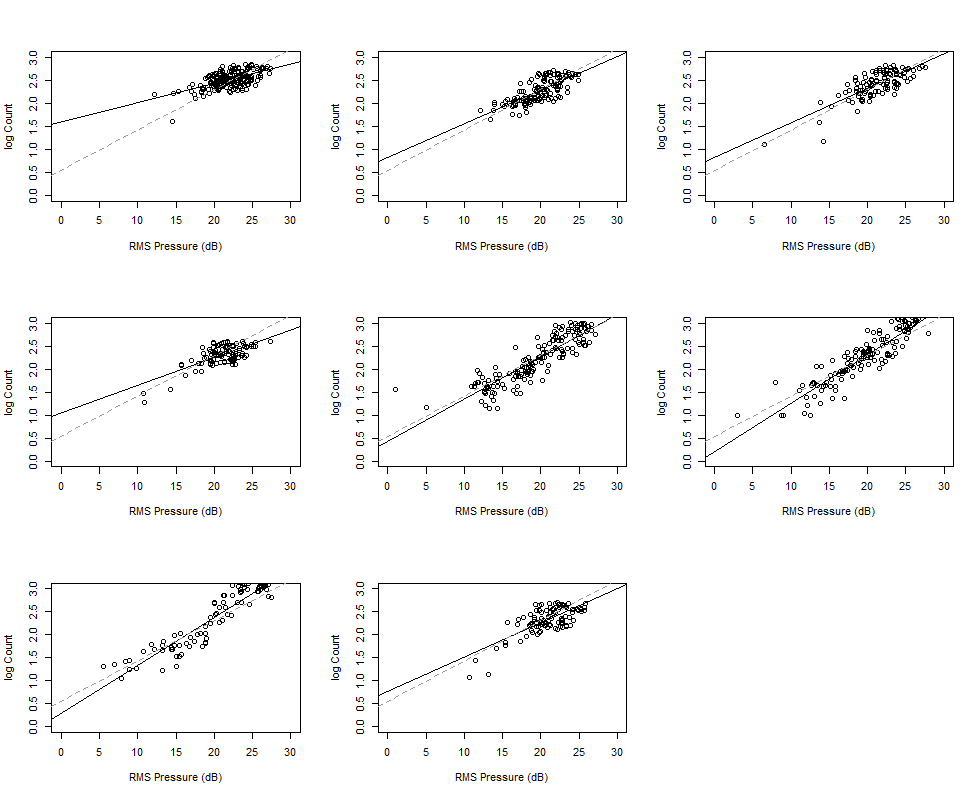


Figure S3. Comparison of RMS pressure to log count for each recorded night at the South Cave. The solid black line represents the regression model for each day, and the dashed line represents the regression model for all days combined (i.e. solid red in Figure 1 in the main article). The distribution of emergence density varied across nights, potentially leading to the AP*day interaction in ANCOVA models.

Supplemental video:

Video V1: Sample video demonstrating the contrast of the bats against the sky during emergence. One frame every 10 s was extracted from the video and bats were counted using ImageJ.
