# Supplementary figures and images for: Estimating colony sizes of emerging bats using acoustic recordings
Source: R Soc Open Sci. 2016 Mar 9;3(3):160022. doi: 10.1098/rsos.160022 (PMC4821278; doi:10.1098/rsos.160022)

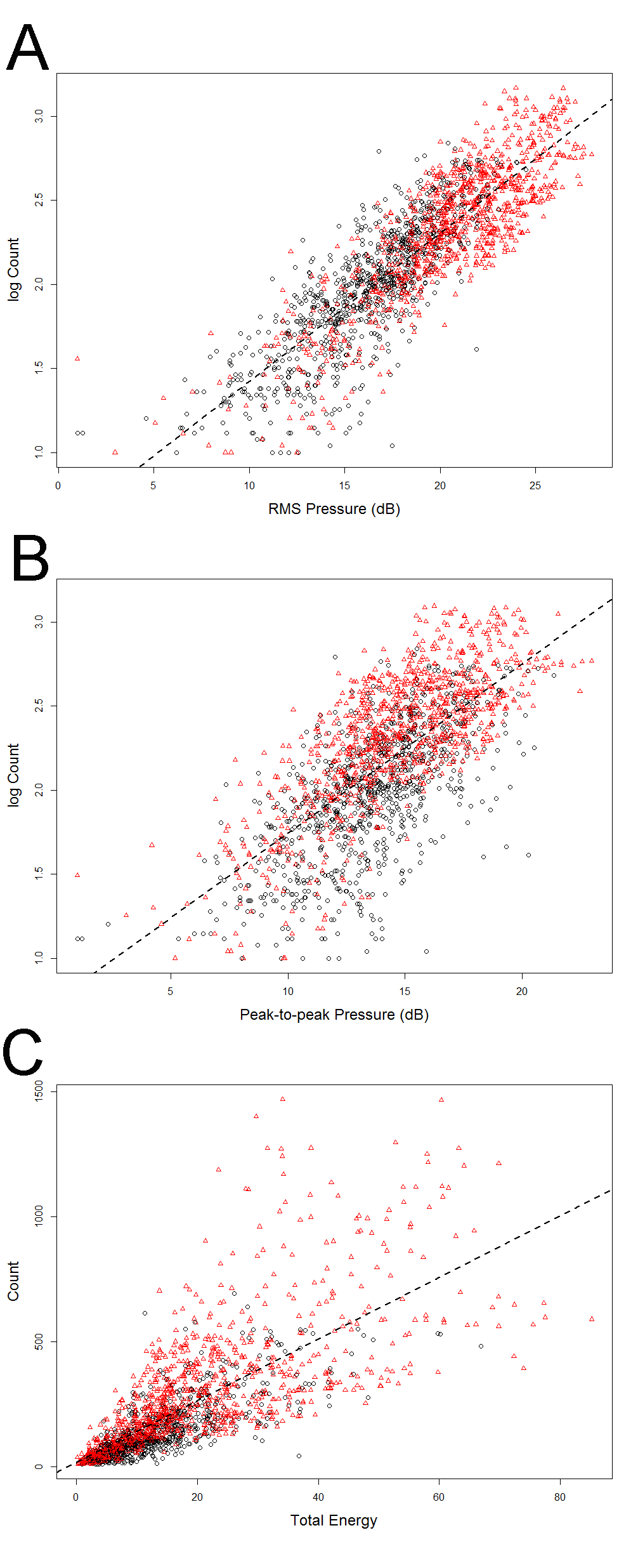

Supplement: Figure S1 [file rsos160022supp2.tif]
